# An interpretable machine learning model integrating mucosal and cellular immunity to predict SARS-CoV-2 reinfection: a multicenter cohort study

**DOI:** 10.1080/22221751.2026.2746253

**Published:** 2026-10-21

**Authors:** Jinqiu Ran, Wen Zhang, Wanying Zhang, Hui Huang, Lejun Gong, Shengxia Yin, Yu Chen, Aofan Wang, Huijin Min, Liguo Zhu, Jinjin Chu, Yuanfang Qin, Rui Huang, Chao Wu, Yuxin Chen

**Affiliations:** a Department of Infectious Disease, Nanjing Drum Tower Hospital, Affiliated Hospital of Medical School, Nanjing University, Nanjing, People's Republic of China; b Department of Laboratory Medicine, Nanjing Drum Tower Hospital, Joint Institute of Nanjing Drum Tower Hospital for Life and Health, College of Life Science, Nanjing Normal University, Nanjing, People's Republic of China; c Department of Laboratory Medicine, Nanjing Drum Tower Hospital Clinical College of Nanjing Medical University, Nanjing, People's Republic of China; d Center of Medical Big Data, Nanjing Drum Tower Hospital, Affiliated Drum Tower Hospital, Medical School of Nanjing University, Nanjing, People's Republic of China; e School of Computer Science, Nanjing University of Posts and Telecommunications, Nanjing, People's Republic of China; f Department of Laboratory Medicine, Nanjing Drum Tower Hospital, Clinical College of Jiangsu University, Nanjing, People's Republic of China; g Department of Acute Infectious Disease Control and Prevention, Jiangsu Provincial Center for Disease Control and Prevention, Nanjing, People's Republic of China; h Department of Laboratory Medicine, Nanjing Drum Tower Hospital, Affiliated Hospital of Medical School, Nanjing University, Nanjing, People's Republic of China

**Keywords:** SARS-CoV-2, reinfection, predictors, machine learning, mucosal immunity, cellular immunity

## Abstract

SARS-CoV-2 reinfection remains a significant public health challenge that requires accurate risk assessment across populations. Using prospectively collected data from two independent cohorts, we developed and externally validated an interpretable machine learning model for predicting symptomatic SARS-CoV-2 reinfection. The cohort comprised 1,156 adults from Wuxi and Nantong, China, with a median follow-up duration of 10 months. Reinfection occurred in 128 participants (11.1%). The model integrated eighteen clinical and immune features, and ultimately identified spike-specific nasal secretory immunoglobulin A (sIgA) and spike-specific CD4^+^ central memory T cells (T_CM_) as key predictors. Developed using the extreme gradient boosting (XGB) algorithm, the prediction model achieved an AUC of 0.835 in the internal validation cohort and demonstrated strong discrimination in the external validation cohort. SHAP analysis revealed that higher nasal spike-specific sIgA was associated with lower risk, while spike-specific CD4^+^ T_CM_ cells exhibited a non-linear association with reinfection. This interpretable immune-based model provides a framework for immune-informed risk stratification, enabling better prediction and management of SARS-CoV-2 reinfection risk in settings with heterogeneous population immunity.

## Introduction

Despite large-scale global vaccination efforts, continuous viral mutations and immune evasion have shifted COVID-19 into an endemic disease characterized by widespread reinfections and seasonal surges that persistently threaten vulnerable populations [1]. Identifying individuals at increased risk of reinfection is therefore important for refining risk stratification and informing targeted preventive strategies. However, protection against SARS-CoV-2 is mediated by a coordinated immune network rather than by any single immune marker alone [2].

Accumulating evidence suggests that mucosal, humoral, and cellular immune responses contribute differently to protection against SARS-CoV-2 infection. Secretory immunoglobulin A (sIgA), the dominant immunoglobulin at the respiratory mucosal surface [3], plays a critical role in blocking viral entry at the site of initial exposure and has been associated with protection against Omicron infection [4]. Circulating antibodies provide systemic immune protection, but their protective effects may be attenuated over time and further compromised by antigenic drift in emerging variants [5,6]. In contrast, cellular immune memory, particularly antigen-specific CD4^+^ and CD8^+^ T cell responses, is thought to contribute to more durable and cross-reactive protection [7,8]. Among these, specific T cell subsets may provide additional insight into immune status. Central memory T cells (T_CM_) localize to secondary lymphoid organs via CCR7-mediated homing, enabling them to effectively support rapid recall responses and secrete effector cytokines upon various stimuli [9–11]. Together, these findings indicate that reinfection risk may be shaped by a multidimensional immune landscape integrating mucosal and cellular immune memory.

However, most previous studies have focused on individual immune correlates rather than integrating multiple immune dimensions into an individualized prediction framework. Traditional statistical approaches may be insufficient to capture the complex and potentially non-linear relationships among immune variables, especially when mucosal, humoral, and cellular features coexist and interact. Machine learning (ML) provides an opportunity to model such multidimensional data and may improve individualized risk prediction when paired with interpretable analytical approaches [12,13].

In this study, we developed and externally validated an interpretable machine learning model for predicting symptomatic SARS-CoV-2 reinfection using baseline clinical and immunological data prospectively collected from two cohorts. By integrating demographic, clinical, and antigen-specific immune features, we aimed to identify key immune predictors associated with reinfection susceptibility and to establish an interpretable framework for individualized reinfection risk stratification and to explore whether integrating mucosal and cellular immune readouts could improve clinically relevant prediction beyond conventional single-marker approaches.

## Methods

### Study cohort and sample collection

This study developed and externally validated a clinical prediction model utilizing prospectively collected data from two community-based cohorts. Between May 2024 and February 2025, a total of 1,186 participants were enrolled in Wuxi and Nantong, China. The study was approved by the Ethics Committee of Nanjing Drum Tower Hospital, and all participants provided written informed consent prior to enrolment. Baseline demographic and clinical data were collected at study entry via structured questionnaires, including age, sex, body mass index, smoking status, drinking status, diabetes status, prior SARS-CoV-2 infection, and COVID-19 vaccination history. Simultaneously, venous blood and nasal mucosal samples were obtained for subsequent immune profiling. The primary outcome was symptomatic SARS-CoV-2 reinfection prospectively ascertained during follow-up, defined as the occurrence of COVID-19-related symptoms accompanied by a positive SARS-CoV-2 antigen or nucleic acid test after enrolment.

Nasal mucosal samples were collected by trained community healthcare workers using the expanding sponge method [14]. Briefly, a polyvinyl alcohol sponge (Beijing Yingjia, China) was expanded in 50 mL of saline, placed in a 10 mL syringe, and compressed to the 4 mL mark to standardize residual volume. The sponge was divided into six equal segments. One segment was inserted into the right nostril for 5 minutes, then immediately transferred upon removal into 1.5 mL of Universal Transport Medium (Copan Diagnostics). Within 4 hours, the fluid was expelled and centrifuged at 1000 rpm for 3 minutes at room temperature. The supernatant was aliquoted and stored at −80°C for sIgA detection.

### Laboratory tests

Peripheral blood mononuclear cells (PBMCs) were isolated from heparinized venous blood by density gradient centrifugation using Lymphoprep™ (Stemcell Technologies, Canada), followed by two washes with phosphate-buffered saline and resuspension in complete RPMI 1640 medium (Thermo Fisher Scientific, USA) supplemented with 10% fetal bovine serum (FBS), 1% penicillin-streptomycin, and 2 mM L-glutamine for immunophenotyping assays.

Given that the JN.1 variant was the predominant circulating SARS-CoV-2 strain during the study period, the JN.1 spike protein was used as both the stimulating antigen and the coating antigen for immune assays. Antigen-specific memory B cells were identified using fluorescently labelled probes targeting the SARS-CoV-2 JN.1 spike receptor-binding domain. Activation-induced marker assays were used to quantify spike-specific circulating T follicular helper (cTfh) cells, and spike-specific CD4^+^ and CD8^+^ T cell subsets, including CD4^+^ T cells with T_CM_, effector memory T cells (T_EM_), and terminally differentiated effector memory T cells re-expressing CD45RA (T_EMRA_). Detailed antibody panels and gating strategies are provided in the Supplementary Methods.

Serum SARS-CoV-2 spike-specific immunoglobulin G (IgG) levels were measured using a chemiluminescent immunoassay (CLIA) kit (Beijing Wantai Biological Pharmacy Enterprise Co., Ltd., Beijing, China) [15]. Nasal SARS-CoV-2 spike-specific sIgA levels were measured by an in-house ELISA [16]. Detailed experimental procedures are provided in Supplementary Methods.

### Dataset preparation and model training

Participants from the Wuxi site (*n* = 570) were randomly divided into a training cohort (*n* = 400) and an internal validation cohort (*n* = 170) using a 7:3 stratified split according to reinfection status. Participants from the Nantong site (*n* = 586) were used as an independent external validation cohort. A total of 18 demographic, clinical, and immune variables were considered for model development. For final model construction, feature selection was performed using the Boruta algorithm in the training cohort only. The Boruta algorithm was run using a random forest classifier with the following parameters: number of trees (ntree) = 500, maximum iterations (maxRuns) = 100, and significance threshold (*p*Value) = 0.01. To assess feature selection stability, Boruta was run on 100 subsamples (80% of the training cohort drawn without replacement), and the frequency of each variable being classified as Confirmed, Tentative, or Rejected was calculated. The variables retained by Boruta were subsequently entered into six machine learning models, including adaptive boosting (ADB), Gaussian naive Bayes (GNB), k-nearest neighbours (KNN), logistic regression (LR), random forest (RF), and extreme gradient boosting (XGB). For models requiring preprocessing, centreing and scaling were performed using parameters estimated from the training cohort and then applied to the validation cohorts. To obtain optimism-corrected performance estimates, a nested 5-fold cross-validation framework was implemented for model tuning and evaluation, in which Boruta feature selection and hyperparameter tuning were strictly restricted to the training folds of each loop. The area under the receiver operating characteristic curve (AUC) was utilized as the primary optimization metric. The final models were then evaluated in the internal and external validation cohorts. SHapley Additive exPlanations (SHAP) values were used to improve model transparency and interpret the contribution of individual features to model predictions. The overall study workflow is shown in Figure S1.

### Statistical analysis

Continuous variables were assessed for normality using the Shapiro-Wilk test. Normally distributed variables were expressed as mean ± standard deviation (SD), and were compared using independent samples t-test. Non-normally distributed variables were presented as median (interquartile range [IQR]) and were compared using the Mann-Whitney U test. Categorical variables were presented as counts (percentages) and were compared using the Chi-square test or Fisher's exact test, as appropriate. A two-side *p* value of < 0.05 was considered statistically significant. All statistical analyses were performed using R software (version 4.3.1).

ML analyses were performed in R version 4.3.1 using the caret package (version 7.0-1). Model discrimination was assessed in the training, internal validation, and external validation cohorts using receiver operating characteristic analysis, and AUCs were calculated accordingly [17,18]. Accuracy, sensitivity, specificity, positive predictive value (PPV), negative predictive value (NPV), and F1 scores were obtained from confusion matrices. Calibration curves were used to assess agreement between predicted probabilities and observed event rates, and decision curve analysis (DCA) was performed to evaluate the potential clinical utility of each model across a range of threshold probabilities [19,20]. To improve interpretability, SHAP values were calculated for all six models [21]. Global feature importance was summarized by mean absolute SHAP values and visualized with beeswarm plots. SHAP dependence plots were further used to show the associations between biomarker values and their contributions to predicted risk.

## Results

### Participant characteristics

Of the 1,186 participants initially enrolled, 30 (2.5%) were lost to follow-up (Table S1): 14 could not be contacted, 7 relocated outside the study area, 4 died from unrelated medical conditions, and 5 discontinued participation for other health-related or personal reasons. There were no significant differences in baseline demographic, clinical, or immunological characteristics between the retained participants and those lost to follow-up (Table S2). Among the remaining 1,156 participants who completed follow-up, 128 (11.1%) experienced SARS-CoV-2 reinfection during a median follow-up of 10 months. The median age was 55 years in participants with reinfection compared with 62 years in those without reinfection, although this difference did not reach statistical significance (*p* = 0.059). No significant differences were observed between the two groups with respect to sex, body mass index (BMI), smoking status, drinking status, and diabetes, number of previous SARS-CoV-2 infections, or number of COVID-19 vaccine doses. At baseline, participants with reinfection had significantly lower levels of spike-specific nasal sIgA titres (4.99 vs. 66.16, *p* < 0.001), spike-specific IgG (113.42 vs.166.52 AU/mL, *p* = 0.001), and spike-specific CD4^+^ T_CM_ cells (0.01% vs. 0.05%, *p* < 0.001) than those without reinfection. In contrast, no significant between-group differences were observed for spike-specific memory B cells, spike-specific cTfh cells, or most other spike-specific T cell subsets. Detailed baseline characteristics are shown in Table 1.

**Table 1.** Baseline characteristics of participants with and without SARS-CoV-2 reinfection.

Variables	Total (*n* = 1156)	Non-reinfection group (*n* = 1028)	Reinfected group (*n*=128)	*p* value
Age (years), median (IQR)	62 (43,72)	62 (44,72)	55 (36, 71)	0.059
Sex, n (%)				0.998
Male	542 (46.9)	482 (46.9)	60 (46.9)	
Female	614 (53.1)	546 (53.1)	68 (53.1)	
Smoking, n (%)				0.198
No	916 (79.2)	809 (78.7)	107 (83.6)	
Yes	240 (20.8)	219 (21.3)	21 (16.4)	
Drinking, n (%)				0.942
No	852 (73.7)	758 (73.7)	94 (73.4)	
Yes	304 (26.3)	270 (26.3)	34 (26.6)	
Diabetes, n (%)				0.174
No	1024 (88.6)	906 (88.1)	118(92.2)	
Yes	132 (11.4)	122 (11.9)	10 (7.8)	
Number of COVID-19 infections, median (IQR)	1 (1,1)	1 (1, 2)	1 (1,1)	0.08
Number of COVID-19 vaccinations, median (IQR)	3 (3,3)	3 (3,3)	3 (3,3)	0.497
BMI, median (IQR)	23.9 (21.6, 26.0)	23.9 (21.6, 26.0)	24.3 (22.1, 26.6)	0.079
Nasal sIgA titres, GMT (95% CI)	49.70 (43.68, 56.54)	66.16 (58.23, 75.19)	4.99 (3.48, 7.15)	<0.001
Serum IgG (AU/mL), GMT (95% CI)	159.59 (149.70, 170.14)	166.52 (155.84, 177.94)	113.42 (91.12, 141.19)	0.001
Memory B cells (%), median (IQR)	1.11 (0.51, 1.89)	1.11 (0.51, 1.85)	1.11 (0.56, 1.97)	0.814
cTfh cells (%), median (IQR)	0.64 (0.33, 1.11)	0.65 (0.34, 1.12)	0.56 (0.28, 1.07)	0.179
CD4^+^ T_CM_ cells (%), median (IQR)	0.05 (0.01, 0.19)	0.05 (0.02, 0.20)	0.01 (0.01, 0.03)	<0.001
CD4^+^ T_EM_ cells (%), median (IQR)	0.82 (0.48, 1.24)	0.83 (0.49, 1.24)	0.81 (0.44, 1.21)	0.606
CD4^+^ T_EMRA_ cells (%), median (IQR)	0.14 (0.07, 0.25)	0.14 (0.07, 0.25)	0.13 (0.07, 0.23)	0.743
CD8^+^ T_CM_ cells (%), median (IQR)	0.03 (0.01, 0.06)	0.03 (0.01, 0.06)	0.03 (0.01, 0.09)	0.214
CD8^+^ T_EM_ cells (%), median (IQR)	0.56 (0.28, 0.94)	0.56 (0.28, 0.93)	0.52 (0.29, 0.97)	0.855
CD8^+^ T_EMRA_ cells (%), median (IQR)	0.44 (0.20, 0.74)	0.43 (0.19, 0.75)	0.47 (0.22, 0.71)	0.627

Note: Data are presented as *n* (%) for categorical variables, median (IQR) for non-normally distributed continuous variables, and GMT (95% CI) for antibody titres. Abbreviations: IQR, interquartile range; CI, confidence interval; GMT, geometric mean titre. BMI, body mass index; sIgA, secretory immunoglobulin A; IgG, immunoglobulin G; cTfh, circulating follicular helper T cells; T_CM_, central memory T cells; T_EM_, effector memory T cells; T_EMRA_, terminally differentiated effector memory T cells.

Baseline demographic and immunological characteristics of the training (n = 400), internal validation (*n* = 170), and external validation (*n* = 586) cohorts are summarized in Table S3. Across the three cohorts, sex, smoking status, and diabetes prevalence were generally comparable (all *p* ≥ 0.05), whereas age, body mass index, drinking status, previous SARS-CoV-2 infection history, and vaccination history differed significantly (all *p* < 0.05). Among the immunological markers, spike-specific CD4^+^ T_EM_ cells, spike-specific CD8^+^T_CM_ cells, spike-specific CD8^+^ T_EM_ cells, and spike-specific CD8^+^ T_EMRA_ cells did not differ significantly across the three cohorts (all *p* ≥ 0.05). In contrast, significant differences were observed for spike-specific nasal sIgA, spike-specific serum IgG, spike-specific memory B cells, spike-specific cTfh cells, spike-specific CD4^+^ T_CM_ cells, and spike-specific CD4^+^ T_EMRA_ cells (all *p* < 0.05).

### Boruta-based feature selection

Our analysis incorporated 18 clinical and immunological parameters for assessment: age, sex, smoking status, drinking status, BMI, diabetes, number of prior SARS-CoV-2 infections, number of COVID-19 vaccinations, spike-specific nasal sIgA, spike-specific serum IgG, spike-specific memory B cells, spike-specific cTfh cells, spike-specific CD4^+^ T_CM_ cells, spike-specific CD4^+^ T_EM_ cells, spike-specific CD4^+^ T_EMRA_ cells, spike-specific CD8^+^ T_CM_ cells, spike-specific CD8^+^ T_EM_ cells, and spike-specific CD8^+^ T_EMRA_ cells. By applying the Boruta feature selection algorithm to our training cohort, we identified SARS-CoV-2 spike-specific nasal sIgA and spike-specific CD4^+^ T_CM_ cells as confirmed key predictors (yellow boxes), which demonstrated the highest variable importance (Z-scores) in predicting SARS-CoV-2 reinfection risk. Additionally, BMI was classified as a tentative factor (blue box), suggesting a potential but less definitive association with reinfection risk. The remaining variables fell into the rejected category (grey boxes), indicating they are unimportant factors for the prediction model (Figure 1). Stability analysis via subsampling confirmed the robustness of our key predictors, nasal sIgA and CD4^+^ T_CM_ cells, which were classified as Confirmed features in 100% of subsampling iterations. In contrast, BMI showed intermediate selection stability, selected as Confirmed in 10% and Tentative in 47% of runs. Other variables showed lower selection stability, with vaccination status rejected in 87% of subsamples, while clinical covariates including sex (97% rejected), drinking (99% rejected), smoking, diabetes, and prior infection history (all 100% rejected) showed limited evidence of consistent feature selection (Table S4).

**Figure 1.**
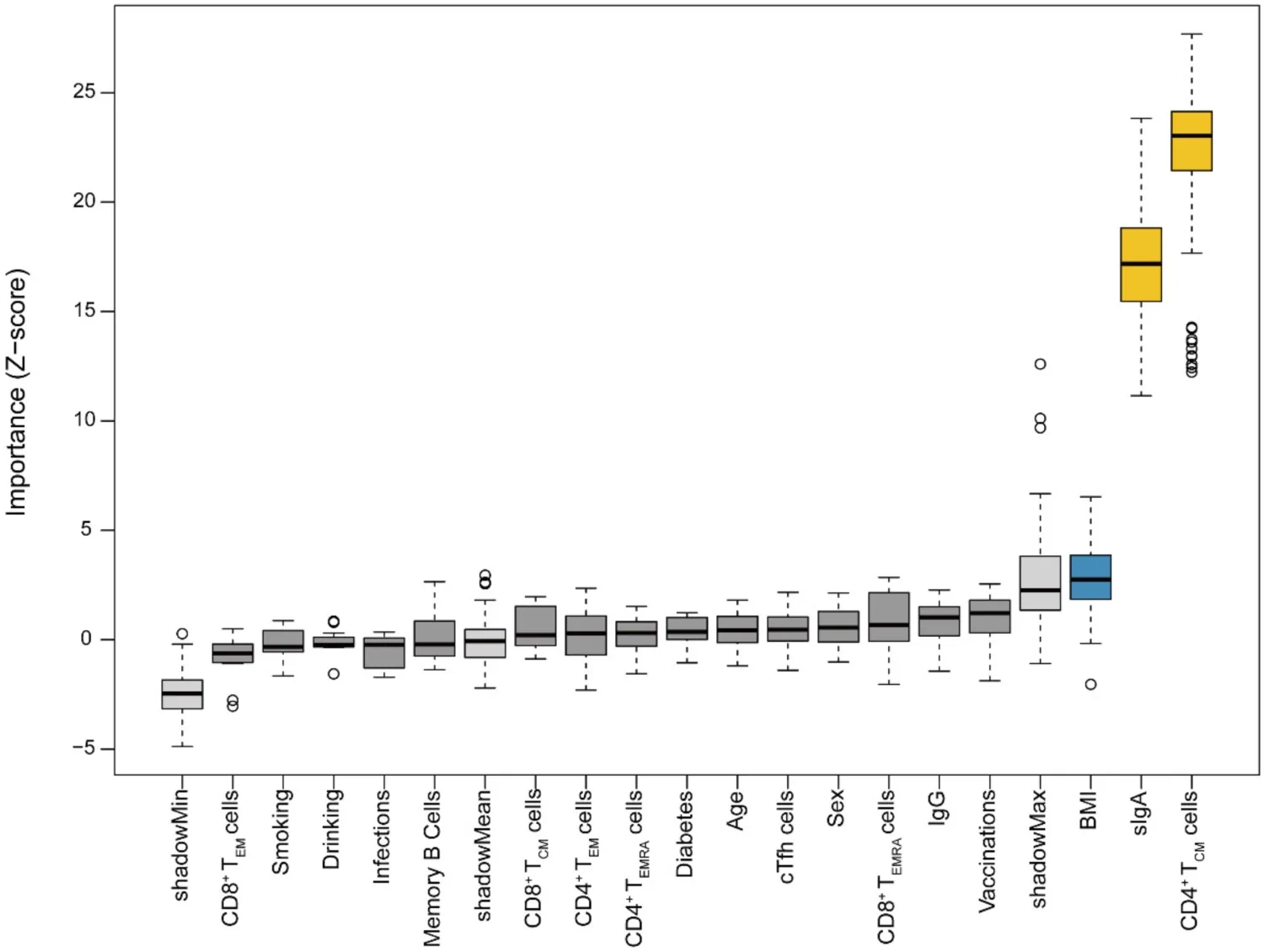
Importance of potential risk factors for SARS-CoV-2 reinfection ranked by the Boruta algorithm. The horizontal axis represents each candidate variable, and the vertical axis represents the Z-score of variable importance. The box plot shows the distribution of Z-scores obtained during Boruta iterations. Yellow boxes indicate confirmed important variables, blue boxes indicate tentative variables, and grey boxes indicate rejected variables. Feature selection stability across 100 subsampling iterations is summarized in Table S4. Abbreviations: BMI, body mass index; sIgA, secretory immunoglobulin A; IgG, immunoglobulin G; cTfh, circulating follicular helper T cells; T_CM_, central memory T cells; T_EM_, effector memory T cells; T_EMRA_, terminally differentiated effector memory T cells.

To assess the robustness of the identified immunological predictors after adjustment for potential clinical confounders, we performed multivariable logistic regression analyses (Table S5). After adjusting for age, sex, infection history, vaccination history, alcohol consumption, smoking, BMI, and diabetes, nasal sIgA remained significantly associated with reduced reinfection risk (OR = 0.995, 95% CI: 0.993–0.997, *p* < 0.001), while CD4⁺ T_CM_ cells showed a consistent protective trend (OR = 0.427, 95% CI: 0.167–1.093, *p* = 0.076), suggesting that the predictive contribution of these immune markers was not substantially explained by baseline clinical characteristics.

### Performance of machine learning models

Based on the two key immune predictors screened by the Boruta algorithm, we evaluated the discrimination performance of six classic ML models for predicting SARS-CoV-2 reinfection risk (Figure 2, Table S6). In the training cohort, the apparent AUC values of the models ranged from 0.783 to 0.957, with the XGB model achieving an apparent AUC of 0.914. To obtain an optimism-corrected estimate of model performance, a nested 5-fold cross-validation was performed, yielding a corrected mean outer-loop AUC of 0.834 ± 0.084 for the XGB model (Table S7). Although KNN and RF displayed higher training AUCs, XGB demonstrated consistently strong discrimination across validation analyses, achieving an AUC of 0.835 in the internal validation cohort, closely aligning with its nested cross-validation estimate. In the external validation cohort, XGB achieved the highest AUC of 0.996 among the evaluated models. Based on its overall discrimination across the training and validation cohorts, XGB was selected as the final prediction model. To comprehensively evaluate model performance, we further analysed the accuracy, sensitivity, and specificity across the three cohorts (Table S6). Notably, in the external validation cohort, the XGB model achieved an accuracy of 0.974, a sensitivity of 1.000, and a specificity of 0.971. Additionally, it yielded a PPV of 0.805, an NPV of 1.000, and an F1 score of 0.892.

**Figure 2.**
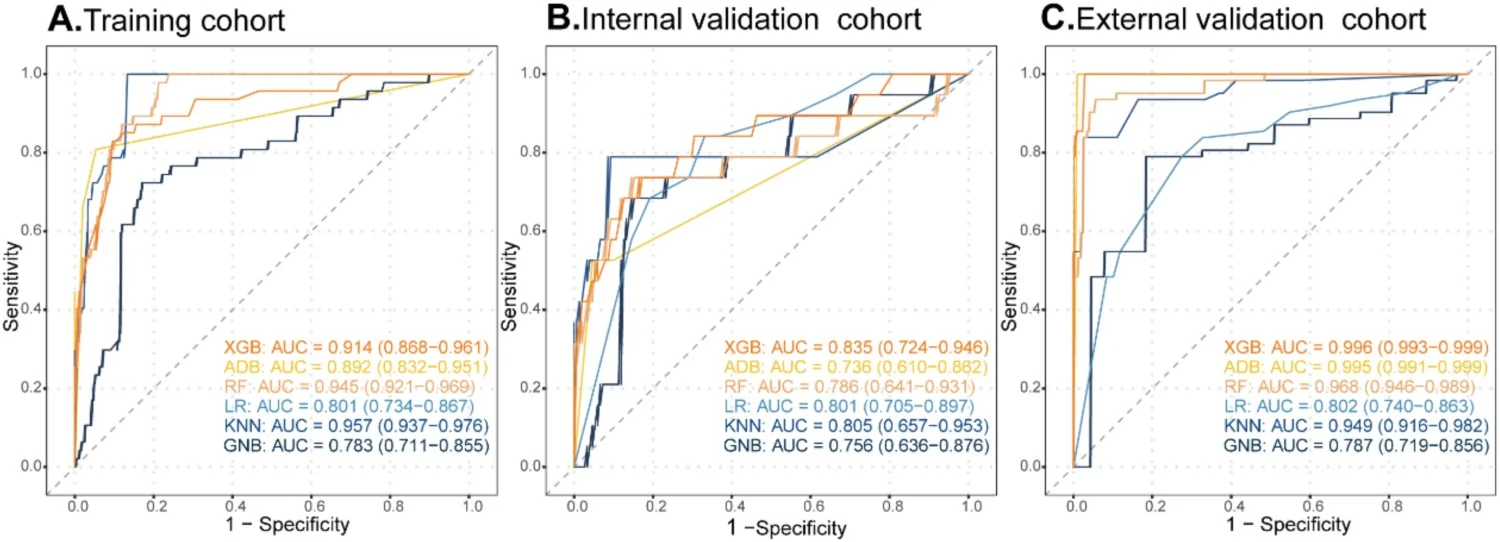
ROC curves of the machine learning models. (A) Training cohort. (B) Internal validation cohort. (C) External validation cohort. Abbreviations: XGB, extreme gradient boosting; ADB, adaptive boosting; RF, random forest; LR, logistic regression; KNN, K-nearest neighbours; GNB, Gaussian naive Bayes.

Furthermore, we examined the individual-level distributions and joint feature spaces of nasal sIgA and CD4^+^ T_CM_ cells (Figure S2). Participants without reinfection in both cohorts generally exhibited higher levels of both markers than those with reinfection (Figure S2A, B). However, the degree of class separation varied between cohorts. While the Wuxi cohort showed moderate overlap between groups (Figure S2C), the Nantong cohort demonstrated greater separation between participants with and without reinfection, with reinfected participants tending to cluster in the lower-left region of the joint feature space (Figure S2D).

### Evaluation of machine learning models

The DCA was performed to evaluate the clinical utility of six ML models. The results demonstrated that the XGB model provided net clinical benefits across a wide range of threshold probabilities in the training, internal validation, and external validation cohorts (Figure 3). Calibration plots further demonstrated good agreement between predicted and observed risks for the XGB model across all three cohorts (Figure S3). Collectively, these multidimensional evaluations establish XGB as the most reliable predictive model in this study.

**Figure 3.**
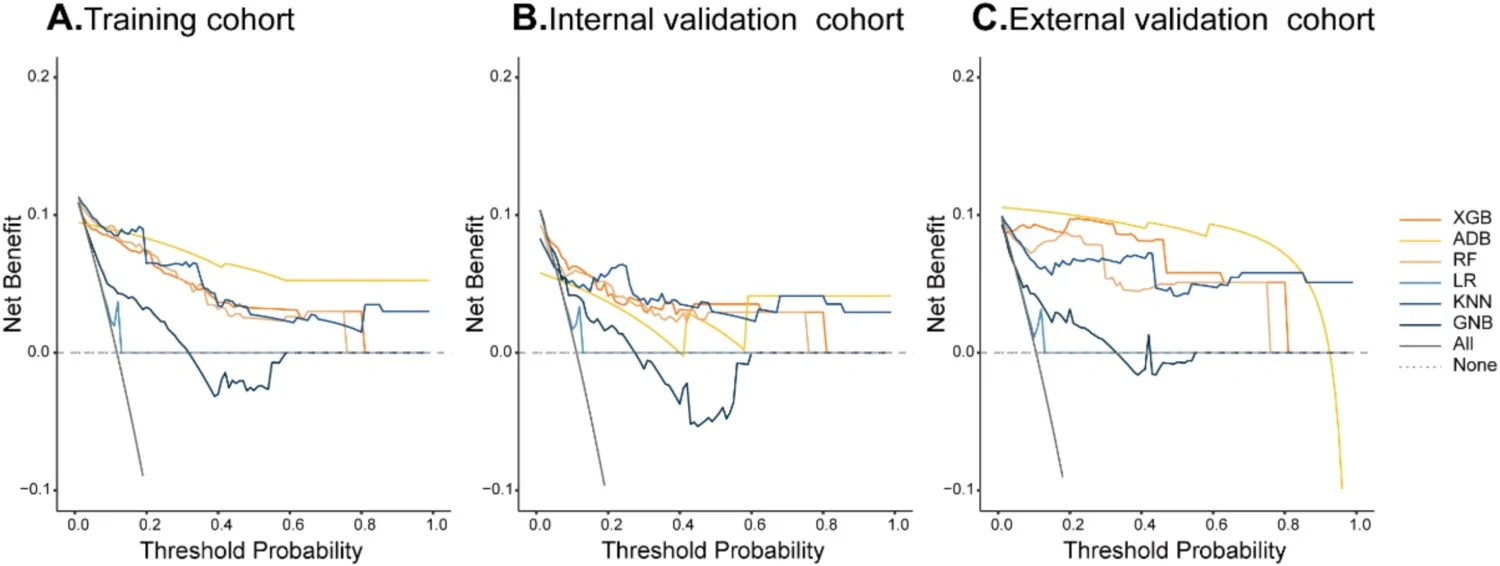
DCA of the machine learning models. (A) Training cohort. (B) Internal validation cohort. (C) External validation cohort. Abbreviations: XGB, extreme gradient boosting; ADB, adaptive boosting; RF, random forest; LR, logistic regression; KNN, K-nearest neighbours; GNB, Gaussian naive Bayes.

### Interpretation of the model

To improve interpretability, SHAP analysis was applied to all models. As shown in Figure 4, in the XGB model, the features with the greatest impact on the prediction results were, in order of importance, spike-specific CD4^+^ T_CM_ cells and spike-specific nasal sIgA in the external validation set. SHAP dependence plots suggested that higher nasal sIgA values were generally associated with lower model-predicted reinfection risk (Figure 5). Spike-specific CD4^+^ T_CM_ cells showed a non-linear relationship with model output, with moderate levels associated with lower predicted risk, whereas very low or relatively high levels were associated with higher predicted risk (Figure 6).

**Figure 4.**
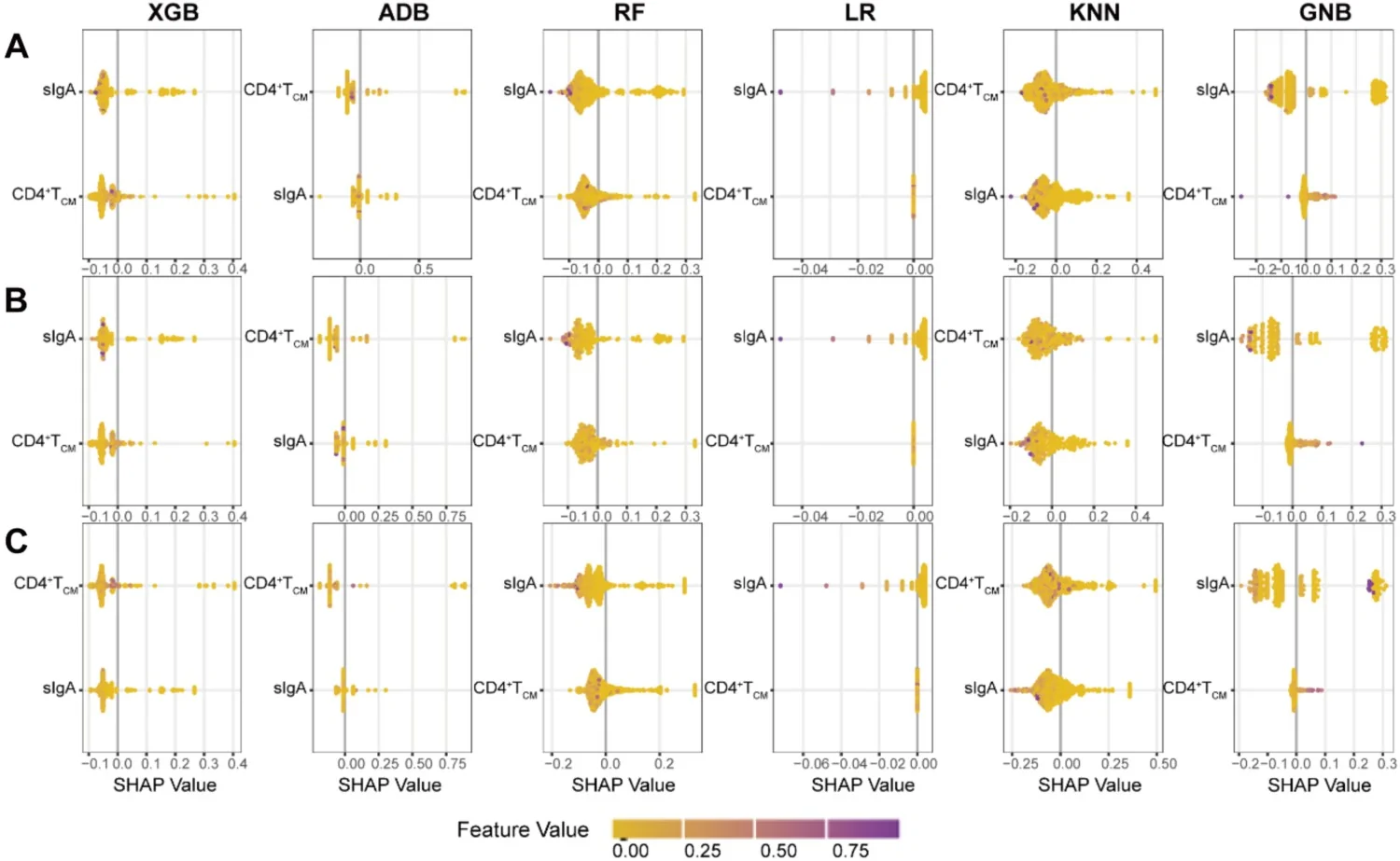
SHAP summary beeswarm plots of the top features of machine learning models. (A) Training cohort. (B) Internal validation cohort. (C) External validation cohort. Each point represents one participant. The x-axis indicates the SHAP value, reflecting the contribution of a given feature to model output, and colour represents the relative feature value from low to high. Abbreviations: XGB, extreme gradient boosting; ADB, adaptive boosting; RF, random forest; LR, logistic regression; KNN, K-nearest neighbours; GNB, Gaussian naive Bayes.

**Figure 5.**
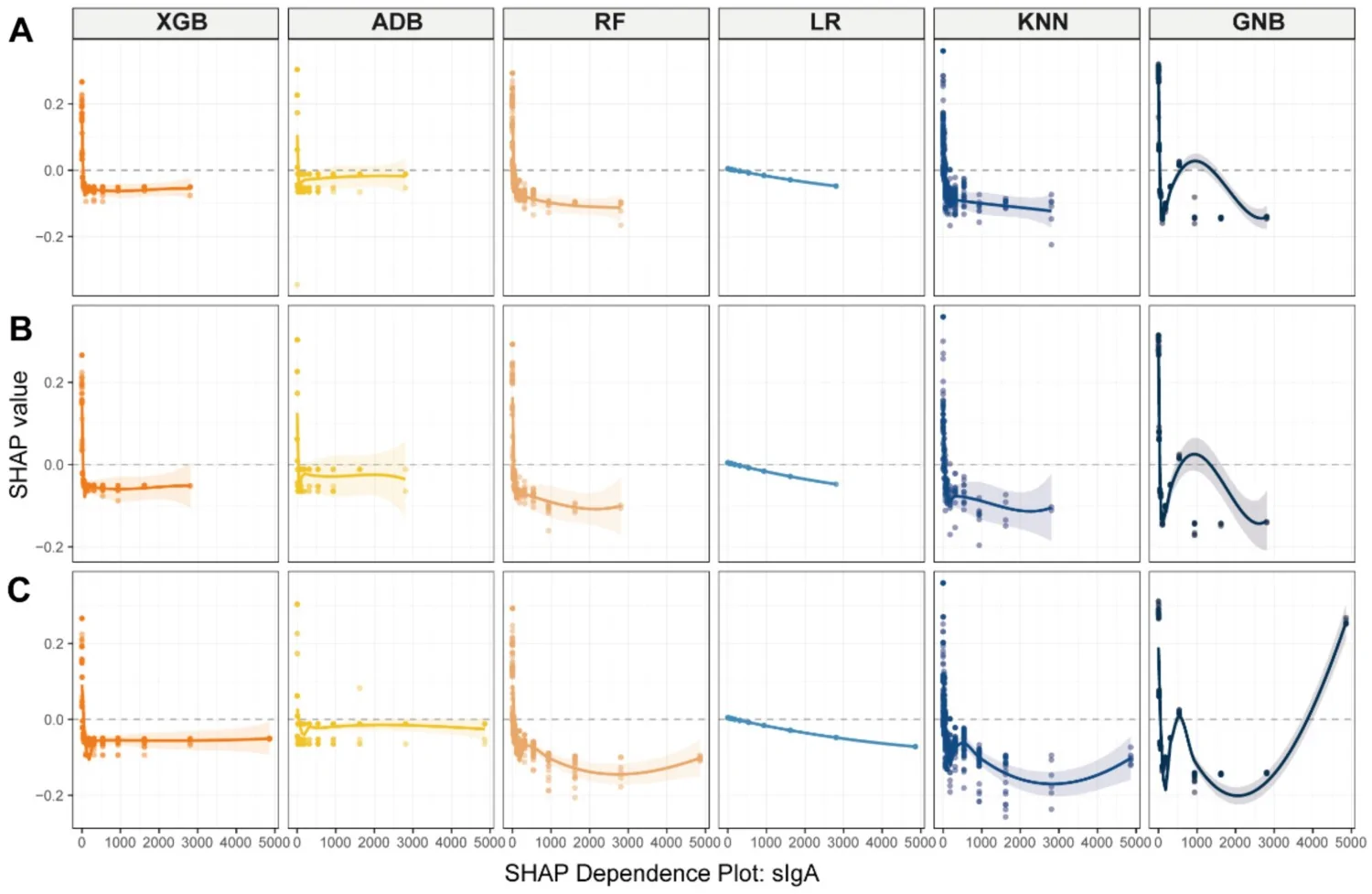
sIgA SHAP dependence plots of machine learning models. (A) Training cohort. (B) Internal validation cohort. (C) External validation cohort. Each point represents one participant. The x-axis shows the measured nasal sIgA value, and the y-axis shows the corresponding SHAP value, indicating the contribution of nasal sIgA to model output. Solid lines with shaded areas illustrate the fitted trend and uncertainty. Abbreviations: XGB, extreme gradient boosting; ADB, adaptive boosting; RF, random forest; LR, logistic regression; KNN, K-nearest neighbours; GNB, Gaussian naive Bayes.

**Figure 6.**
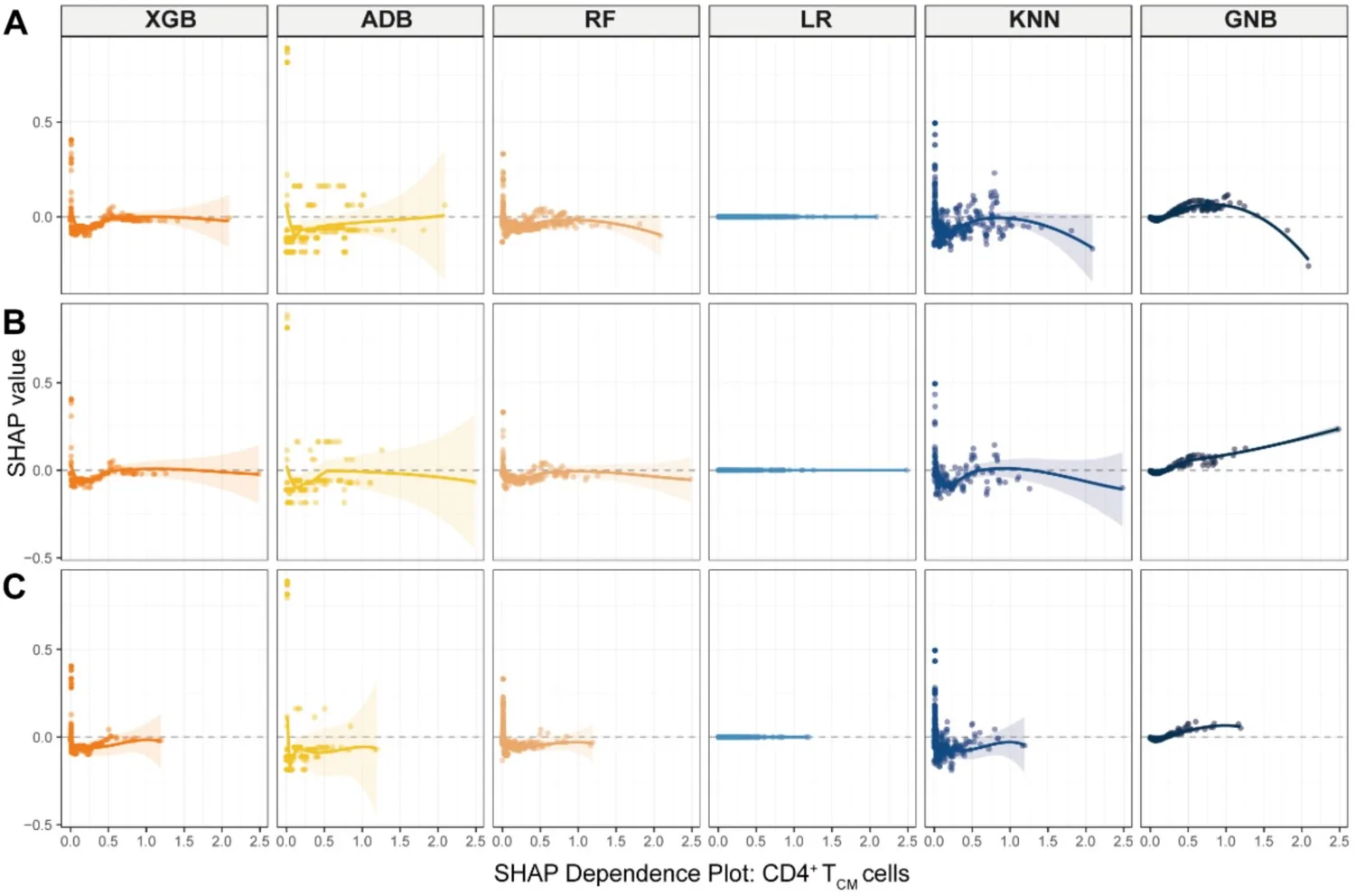
CD4^+^ T_CM_ cell SHAP dependence plots of machine learning models. (A) Training cohort. (B) Internal validation cohort. (C) External validation cohort. Each point represents one participant. The x-axis shows the measured CD4^+^ T_CM_ cells value, and the y-axis shows the corresponding SHAP value, indicating the contribution of CD4^+^ T_CM_ cells to model output. Solid lines with shaded areas illustrate the fitted trend and uncertainty. Abbreviations: XGB, extreme gradient boosting; ADB, adaptive boosting; RF, random forest; LR, logistic regression; KNN, K-nearest neighbours; GNB, Gaussian naive Bayes.

### Development of a web-based application

Based on the final XGB model, we developed a web-based application that allows users to input the values of the two selected immune markers and obtain an estimated risk of SARS-CoV-2 reinfection. The application is available online at: https://www.iskmodel.com/tool/xgb.

## Discussion

In this study using data from two prospective cohorts, we developed and externally validated an interpretable machine learning model to predict SARS-CoV-2 reinfection risk based on baseline immune features, evaluating its performance in an independent validation cohort. Among the six machine learning models evaluated, the XGB model showed the most favourable overall performance across the training and validation cohorts. Importantly, the model consistently highlighted two immune features, spike-specific nasal sIgA and spike-specific CD4^+^ T_CM_ cells, as the most informative predictors. These findings support the concept that reinfection susceptibility is shaped by coordinated mucosal and cellular immune memory rather than by a single systemic marker alone.

The upper respiratory tract serves as the initial site of SARS-CoV-2 infection, making mucosal immunity particularly relevant to protection against reinfection. Nasal sIgA, the dominant immunoglobulin at the respiratory mucosal surface, plays an important role in limiting viral attachment and early invasion [22]. Previous studies have shown that mucosal IgA may retain neutralizing activity against immune-evasive Omicron subvariants and that higher nasal anti-spike IgA levels are associated with reduced risk of subsequent infection [23,24]. Consistent with these observations, our findings suggest that higher levels of spike-specific nasal sIgA are associated with lower model-predicted reinfection risk. This result further supports the potential value of mucosal immune markers in individualized risk stratification.

Our findings also underscore the relevance of antigen-specific CD4^+^ T cell subsets in reinfection risk prediction. Previous studies, including our own, have suggested that durable cellular immune responses may provide protective value even when humoral immunity is limited [25–27]. In the present study, spike-specific CD4^+^ T_CM_ cells emerged as a key model feature, suggesting that differences in the quality and composition of CD4^+^ T cell memory may contribute to heterogeneity in reinfection susceptibility.

We observed a non-linear association between spike-specific CD4^+^ T_CM_ cells and model output, with moderate levels associated with lower predicted reinfection risk, whereas very low or relatively high levels were associated with higher predicted risk. This pattern suggests that spike-specific CD4^+^ T_CM_ cells may serve as an important indicator of the balance between protective immune memory and immune dysregulation. As a key memory subset, CD4^+^ T_CM_ cells retain proliferative capacity and can rapidly differentiate into effector populations upon antigen re-exposure, thereby contributing to recall immunity and limiting reinfection risk [28]. In this context, very low levels of spike-specific CD4^+^ T_CM_ cells may reflect insufficient establishment or maintenance of protective immune memory. By contrast, relatively high levels may not necessarily indicate superior protection. Excessive expansion of the memory compartment may be accompanied by contraction of other T-cell compartments, including the naive T-cell pool, potentially constraining TCR repertoire diversity required for recognition of newly emerging immune-evasive variants [29,30]. In addition, persistently elevated T_CM_ frequencies may reflect repeated or prolonged antigenic stimulation, prior severe immune activation, or heterogeneity in memory quality rather than functionally efficient recall capacity [31]. Therefore, the observed non-linear pattern may reflect differences not only in the quantity of memory T cells but also in their qualitative fitness, activation state, and host immune history.

ML, integrating mathematics, statistics, and computer science, improves disease diagnosis and prognosis by extracting hidden patterns from complex data [32]. ML models have been widely applied in various aspects of COVID-19 research, including viral transmission prediction [33], prognosis assessment [34] and high-risk population identification [35]. In this study, the XGB model demonstrated favourable predictive performance for predicting SARS-CoV-2 reinfection risk. The XGB model is a gradient boosting-based ensemble model that sequentially optimizes weak classifiers through gradient descent, delivering high efficiency and accuracy for structured and large-scale datasets [36]. In our study, XGB appeared to handle the complex and non-linear relationships between immune features and reinfection risk more effectively than the other evaluated algorithms. This finding suggests that interpretable machine learning may provide clinically useful support for reinfection risk stratification when multidimensional immune data are available.

Several limitations of this study warrant mention. First, the study participants were mainly enrolled from the Yangtze River Delta, which may limit the representativeness of the study population. Further validation in broader and more diverse populations is therefore needed. Second, immune markers were assessed only at baseline, and their longitudinal trajectories during follow-up were not captured. Third, relying on symptom-triggered self-testing rather than systematic screening may have introduced ascertainment bias by under-detecting asymptomatic cases, despite free test kits and monthly follow-ups. Consequently, the model is primarily applicable to symptomatic, test-confirmed reinfections, and extending its use to all SARS-CoV-2 infections would require further validation and potential recalibration. Fourth, since our assays utilized the JN.1 spike antigen, the model's predictive performance may not be directly transferable to SARS-CoV-2 variants with substantial antigenic divergence from JN.1. Although mucosal IgA responses and memory T-cell responses may contribute to cross-variant immune protection, their predictive efficacy against future strains with distinct mutations requires further validation. Fifth, heterogeneous histories of prior SARS-CoV-2 infection and vaccination may influence individual immune imprinting, potentially modulating the performance and generalizability of specific immune correlates across different populations. Sixth, although the model demonstrated strong discrimination in an independent external validation cohort, both cohorts were recruited within the same province and during a similar epidemic period. Further evaluation in geographically, temporally, and antigenically distinct populations will help define its broader applicability and facilitate future clinical translation.

In summary, we developed and externally validated an interpretable machine learning model for predicting SARS-CoV-2 reinfection risk using baseline immune features in a multicenter cohort. A two-marker immune signature consisting of spike-specific nasal sIgA and spike-specific CD4^+^ T_CM_ cells showed strong predictive value and provided insight into immune correlates associated with reinfection susceptibility. These findings support the integration of mucosal and cellular immune markers into future risk stratification frameworks and may also inform the development of predictive models for reinfection or breakthrough infection caused by other respiratory viruses.

## Supplementary Material

264321380 Supplementary materials.docx

## References

[1] Wang Q, Iketani S, Li Z, et al. Alarming antibody evasion properties of rising SARS-CoV-2 BQ and XBB subvariants. Cell. 2023;186(2):279–286.e8. doi:10.1016/j.cell.2022.12.018 PMC9747694 36580913

[2] Sette A, Crotty S. Adaptive immunity to SARS-CoV-2 and COVID-19. Cell. 2021;184(4):861–880. doi:10.1016/j.cell.2021.01.007 PMC7803150 33497610

[3] Whitsett JA, Alenghat T. Respiratory epithelial cells orchestrate pulmonary innate immunity. Nat Immunol. 2015;16(1):27–35. doi:10.1038/ni.3045 PMC4318521 25521682

[4] Havervall S, Marking U, Svensson J, et al. Anti-spike mucosal IgA protection against SARS-CoV-2 Omicron infection. N Engl J Med. 2022;387(14):1333–1336. doi:10.1056/NEJMc2209651 PMC9511632 36103621

[5] Corbett KS, Nason MC, Flach B, et al. Immune correlates of protection by mRNA-1273 vaccine against SARS-CoV-2 in nonhuman primates. Science. 2021;373(6561):eabj0299. doi:10.1126/science.abj0299 PMC8449013 34529476

[6] Hachmann NP, Miller J, Collier AY, et al. Neutralization escape by SARS-CoV-2 Omicron subvariants BA.2.12.1, BA.4, and BA.5. N Engl J Med. 2022;387(1):86–88. doi:10.1056/NEJMc2206576 PMC9258748 35731894

[7] Pardieck IN, van der Sluis TC, van der Gracht ETI, et al. A third vaccination with a single T cell epitope confers protection in a murine model of SARS-CoV-2 infection. Nat Commun. 2022;13(1):3966. doi:10.1038/s41467-022-31721-6 PMC9267705 35803932

[8] Ishii H, Nomura T, Yamamoto H, et al. Neutralizing-antibody-independent SARS-CoV-2 control correlated with intranasal-vaccine-induced CD8(+) T cell responses. Cell Rep Med. 2022;3(2):100520. doi:10.1016/j.xcrm.2022.100520 PMC8768424 35233545

[9] Xie L, Fang J, Yu J, et al. The role of CD4(+) T cells in tumor and chronic viral immune responses. MedComm. 2023;4(5):e390. doi:10.1002/mco2.390 PMC10565399 37829505

[10] Sallusto F, Lenig D, Förster R, et al. Two subsets of memory T lymphocytes with distinct homing potentials and effector functions. Nature. 1999;401(6754):708–712. doi:10.1038/44385 10537110

[11] Farber DL, Yudanin NA, Restifo NP. Human memory T cells: generation, compartmentalization and homeostasis. Nat Rev Immunol. 2014;14(1):24–35. doi:10.1038/nri3567 PMC4032067 24336101

[12] Kou Z, Huang YF, Shen A, et al. Prediction of pandemic risk for animal-origin coronavirus using a deep learning method. Infect Dis Poverty. 2021;10(1):128. doi:10.1186/s40249-021-00912-6 PMC8542360 34689829

[13] Ma E, Guo X, Hu M, et al. A predictive language model for SARS-CoV-2 evolution. Signal Transduct Target Ther. 2024;9(1):353. doi:10.1038/s41392-024-02066-x PMC11663983 39710752

[14] Zhang X, Jia J, Hu C, et al. Exploring the standardized detection and sampling methods of human nasal SARS-CoV-2 RBD IgA. Front Immunol. 2025;16:1571418. doi:10.3389/fimmu.2025.1571418 PMC12129971 40463384

[15] Wu S, Hou H, Li H, et al. Comparison of the performance of 24 severe acute respiratory syndrome coronavirus 2 antibody assays in the diagnosis of coronavirus disease 2019 patients. Front Microbiol. 2022;13:876227. doi:10.3389/fmicb.2022.876227 PMC9393512 36003928

[16] Li C, Yu J, Issa R, et al. CoronaVac-induced antibodies that facilitate Fc-mediated neutrophil phagocytosis track with COVID-19 disease resolution. Emerg Microbes Infect. 2025;14(1):2434567. doi:10.1080/22221751.2024.2434567 PMC11731273 39584817

[17] Dutschmann TM, Kinzel L, Ter Laak A, et al. Large-scale evaluation of k-fold cross-validation ensembles for uncertainty estimation. J Cheminform. 2023;15(1):49. doi:10.1186/s13321-023-00709-9 PMC10142532 37118768

[18] Pepe M, Longton G, Janes H. Estimation and comparison of receiver operating characteristic curves. Stata J. 2009;9(1):1. doi:10.1177/1536867X0900900101 PMC2774909 20161343

[19] Vickers AJ, Cronin AM, Elkin EB, et al. Extensions to decision curve analysis, a novel method for evaluating diagnostic tests, prediction models and molecular markers. BMC Med Inform Decis Mak. 2008;8:53. doi:10.1186/1472-6947-8-53. PMC2611975 19036144

[20] Van Calster B, McLernon DJ, van Smeden M, et al. Calibration: the Achilles heel of predictive analytics. BMC Med. 2019;17(1):230. doi:10.1186/s12916-019-1466-7 PMC6912996 31842878

[21] Allgaier J, Mulansky L, Draelos RL, et al. How does the model make predictions? A systematic literature review on the explainability power of machine learning in healthcare. Artif Intell Med. 2023;143:102616. doi:10.1016/j.artmed.2023.102616 37673561

[22] Oh JE, Song E, Moriyama M, et al. Intranasal priming induces local lung-resident B cell populations that secrete protective mucosal antiviral IgA. Sci Immunol. 2021;6(66):eabj5129. doi:10.1126/sciimmunol.abj5129 PMC8762609 34890255

[23] Chen S, Zhang Z, Wang Q, et al. Intranasal adenovirus-vectored Omicron vaccine induced nasal immunoglobulin A has superior neutralizing potency than serum antibodies. Signal Transduct Target Ther. 2024;9(1):190. doi:10.1038/s41392-024-01906-0 PMC11263566 39039046

[24] Marking U, Bladh O, Havervall S, et al. 7-month duration of SARS-CoV-2 mucosal immunoglobulin-A responses and protection. Lancet Infect Dis. 2023;23(2):150–152. doi:10.1016/S1473-3099(22)00834-9 PMC9833832 36640796

[25] Chen Y, Shen H, Huang R, et al. Serum neutralising activity against SARS-CoV-2 variants elicited by CoronaVac. Lancet Infect Dis. 2021;21(8):1071–1072. doi:10.1016/S1473-3099(21)00287-5 PMC8159188 34051887

[26] Chen Y, Yin S, Tong X, et al. Dynamic SARS-CoV-2-specific B-cell and T-cell responses following immunization with an inactivated COVID-19 vaccine. Clin Microbiol Infect. 2022;28(3):410–418. doi:10.1016/j.cmi.2021.10.006 PMC8547974 34715346

[27] Chen Y, Chen L, Yin S, et al. The third dose of CoronVac vaccination induces broad and potent adaptive immune responses that recognize SARS-CoV-2 Delta and Omicron variants. Emerg Microbes Infect. 2022;11(1):1524–1536. doi:10.1080/22221751.2022.2081614 PMC9176682 35608053

[28] Marusina AI, Ono Y, Merleev AA, et al. CD4(+) virtual memory: antigen-inexperienced T cells reside in the naïve, regulatory, and memory T cell compartments at similar frequencies, implications for autoimmunity. J Autoimmun. 2017 Feb;77:76–88. doi:10.1016/j.jaut.2016.11.001 PMC6066671 27894837

[29] Rydyznski Moderbacher C, Ramirez SI, Dan JM, et al. Antigen-specific adaptive immunity to SARS-CoV-2 in acute COVID-19 and associations with age and disease severity. Cell. 2020;183(4):996–1012.e19. doi:10.1016/j.cell.2020.09.038 PMC7494270 33010815

[30] Mathew D, Giles JR, Baxter AE, et al. Deep immune profiling of COVID-19 patients reveals distinct immunotypes with therapeutic implications. Science. 2020;369(6508):eabc8511. doi:10.1126/science.abc8511. PMC7402624 32669297

[31] Bertoletti A, Le Bert N, Tan AT. SARS-CoV-2-specific T cells in the changing landscape of the COVID-19 pandemic. Immunity. 2022;55(10):1764–1778. doi:10.1016/j.immuni.2022.08.008 PMC9385766 36049482

[32] Sidey-Gibbons JAM, Sidey-Gibbons CJ. Machine learning in medicine: a practical introduction. BMC Med Res Methodol. 2019;19(1):64. doi:10.1186/s12874-019-0681-4 PMC6425557 30890124

[33] John CC, Ponnusamy V, Krishnan Chandrasekaran S, et al. A survey on mathematical, machine learning and deep learning models for COVID-19 transmission and diagnosis. IEEE Rev Biomed Eng. 2022;15:325–340. doi:10.1109/RBME.2021.3069213 PMC8905610 33769936

[34] de Fátima Cobre A, Surek M, Stremel DP, et al. Diagnosis and prognosis of COVID-19 employing analysis of patients’ plasma and serum via LC-MS and machine learning. Comput Biol Med. 2022;146:105659. doi:10.1016/j.compbiomed.2022.105659 PMC9123826 35751188

[35] Banoei MM, Dinparastisaleh R, Zadeh AV, et al. Machine-learning-based COVID-19 mortality prediction model and identification of patients at low and high risk of dying. Crit Care. 2021;25(1):328. doi:10.1186/s13054-021-03749-5 PMC8424411 34496940

[36] Zhang W, Pan M, Wang P, et al. Comparative analysis of XGB, CNN, and ResNet models for predicting moisture content in Porphyra yezoensis using near-infrared spectroscopy. Foods. 2024;13(19):3023. doi:10.3390/foods13193023. PMC11475958 39410057

