# Supplementary material for: An interpretable machine learning model integrating mucosal and cellular immunity to predict SARS-CoV-2 reinfection: a multicenter cohort study
Source: Emerg Microbes Infect. 2026 Oct 21;15(1):2746253. doi: 10.1080/22221751.2026.2746253 (PMC13700530; doi:10.1080/22221751.2026.2746253)
Supplement: Supplementary file 1 [file TEMI_A_2746253_SM8660.docx]

**Supplementary Methods**

**Cell stimulation and flow cytometry immunophenotyping**

For B-cell phenotyping, cells were stained with anti-CD19-BV421 (clone HIB19), anti-CD27-BB700 (clone M-T271), and anti-IgD-BV786 (clone IA6-2), and JN.1 spike-specific memory B cells were defined as CD19⁺IgD⁻CD27⁺Probe⁺ using PerCP-Cy5.5-conjugated JN.1 spike RBD probes.

For T-cell stimulation, freshly isolated PBMCs were plated at 1 × 10^6^ cells/well in 96-well plates and stimulated overnight with a SARS-CoV-2 JN.1 spike peptide pool (1 μg/mL) in RPMI 1640 supplemented with 2% fetal calf serum (FCS). After stimulation, cells were stained in DPBS containing 2% FCS for 20 min at 4°C. For cTfh-cell analysis, cells were stained with anti-CD4-PE-Cy7 (clone SK3), anti-CXCR5-BV421 (clone RF8B2), anti-CD134-BV510 (clone L106), and anti-CD137-PE (clone 4B4-1). Spike-specific cTfh cells were defined as CD4⁺CXCR5⁺CD134⁺CD137⁺.

For T-cell subset analysis, cells were stained with anti-CD4-APC-Cy7 (clone RPA-T4), anti-CD8-FITC (clone G42-8), anti-CD134-BV510 (clone L106), anti-CD137-PE (clone 4B4-1), anti-CD69-PE-Cy7 (clone FN50), anti-CD45RA-BV711 (clone HI100), and anti-CCR7-BV650 (clone 2-L1-A). Activated spike-specific CD4⁺ and CD8⁺ T cells were identified using an activation-induced marker (AIM) assay as CD4⁺CD134⁺CD137⁺ and CD8⁺CD69⁺CD137⁺, respectively. Based on CCR7 and CD45RA expression, spike-specific CD4⁺ and CD8⁺ T cell subsets were further classified as central memory T cells (T_CM_ cells, CCR7⁺CD45RA⁻), effector memory T cells (T_EM_ cells, CCR7⁻CD45RA⁻), and terminally differentiated effector memory T cells re-expressing CD45RA (T_EMRA_ cells, CCR7⁻CD45RA⁺).

Following surface staining, samples were acquired on a BD FACS Aria III cytometer (BD Biosciences), and data were processed using FlowJo software (v10.7.1, BD Biosciences). Compensation and gating strategies were established using fluorescence minus one (FMO) controls.

**Measurement of nasal SARS-CoV-2 spike-specific sIgA by ELISA**

SARS-CoV-2 spike-specific secretory IgA (sIgA) in nasal samples was measured using an in-house ELISA. Briefly, 96-well plates were first coated with SARS-CoV-2 JN.1 spike protein (500 ng/mL) and incubated overnight at 4°C. Nasal samples stored at -80°C were thawed and initially diluted 1:20 in assay buffer, followed by two-fold serial dilutions. The diluted samples were added to the coated wells and incubated for 2 h at 37°C. After washing, an HRP-conjugated anti-human IgA secondary antibody (Abcam, UK) was added and incubated for 1 h at 37°C. Tetramethylbenzidine (TMB) substrate was subsequently added, and the color was developed for 5 min before optical density (OD) was measured at 450 nm. The cutoff was determined based on the background OD values of assay buffer blank controls plus three standard deviations. Endpoint titers were defined as the highest sample dilution yielding an OD value above the predefined cutoff.

**Figure S1. Flowchart of study cohort derivation and machine learning model development and validation.** The flowchart illustrates participant selection, cohort allocation, and the machine learning workflow. The Wuxi cohort underwent stratified random splitting (7:3 ratio) into training and internal validation cohorts, whereas the Nantong cohort served as an independent external validation cohort. Within the training cohort, Boruta feature selection and hyperparameter tuning were embedded in a nested 5-fold cross-validation framework to minimize information leakage during resampling and obtain optimism-corrected performance estimates. The final models were subsequently evaluated on both internal and external validation cohorts, followed by SHAP-based model interpretation. Abbreviations: CV, cross-validation; ML, machine learning; SHAP, SHapley Additive exPlanations; XGB, extreme gradient boosting; ADB, adaptive boosting; RF, random forest; LR, logistic regression; KNN, K-nearest neighbors; GNB, Gaussian naive Bayes.

**Figure S2. Distribution and joint feature space of nasal sIgA and CD4^+^ T_CM_ cells by reinfection status.** (A) Distribution of nasal sIgA titers in participants with and without reinfection in the Wuxi (left) and Nantong (right) cohorts, shown as violin-box plots with individual data points. (B) Distribution of CD4^+^ T_CM_ cell frequencies in participants with and without reinfection in the Wuxi (left) and Nantong (right) cohorts, shown as violin-box plots with individual data points. (C) Joint feature space of nasal sIgA titers (x-axis) and CD4^+^ T_CM_ cell frequencies (y-axis) in the Wuxi cohort. (D) Joint feature space of nasal sIgA titers (x-axis) and CD4^+^ T_CM_ cell frequencies (y-axis) in the Nantong cohort. *P*-values were calculated using two-tailed Wilcoxon rank-sum tests. Shaded ellipses in panels C and D represent 90% data ellipses, and diamonds indicate group centroids. Abbreviations: GMT, geometric mean titer; sIgA, secretory immunoglobulin A; T_CM_, central memory T cells.

**Figure S3. Calibration curves of the machine learning models.** Calibration curves showing the agreement between predicted probabilities and observed SARS-CoV-2 reinfection rates in the (A) training cohort, (B) internal validation cohort, and (C) external validation cohort. The dashed diagonal line represents perfect calibration. Points represent the observed event rates versus mean predicted probabilities within quantile-based risk deciles, with point sizes reflecting bin sample sizes. Abbreviations: XGB, extreme gradient boosting; ADB, adaptive boosting; RF, random forest; LR, logistic regression; KNN, K-nearest neighbors; GNB, Gaussian naive Bayes.

**Table S1.** Detailed reasons for loss to follow-up (n = 30)

| **Reason** | **n (%)** |
| --- | --- |
| Unable to contact | 14 (46.7) |
| Relocation outside study area | 7 (23.3) |
| Death (pneumonia: 1; subarachnoid hemorrhage: 1; tumor: 1; lung cancer: 1) | 4 (13.3) |
| Recent surgery | 1 (3.3) |
| Hospitalization for heart failure | 1 (3.3) |
| Extended business travel | 1 (3.3) |
| Acute illness | 1 (3.3) |
| Work-related relocation | 1 (3.3) |

**Table S2.** Baseline characteristics and immunological features of participants retained in the study and those lost to follow-up.

| **Variables** | **Retained Group (n=1,156)** | **Lost to Follow-up Group (n=30)** | ***P* value** |
| --- | --- | --- | --- |
| Age (years), median (IQR) | 62 (43, 72) | 68 (61, 74) | 0.062 |
| Sex, n (%) |  |  | 0.736 |
| Male | 542 (46.9) | 15 (50.0) |  |
| Female | 614 (53.1) | 15 (50.0) |  |
| Smoking, n (%) |  |  | 0.220 |
| No | 916 (79.2) | 21 (70.0) |  |
| Yes | 240 (20.8) | 9 (30.0) |  |
| Drinking，n (%) |  |  | 0.235 |
| No | 852 (73.7) | 25 (83.3) |  |
| Yes | 304 (26.3) | 5 (16.7) |  |
| Diabetes, n (%) |  |  | 0.086 |
| No | 1024 (88.6) | 23 (76.7) |  |
| Yes | 132 (11.4) | 7 (23.3) |  |
| Number of COVID-19 infections, median (IQR) | 1 (1, 2) | 1 (1,1) | 0.932 |
| Number of COVID-19 vaccinations, median (IQR) | 3 (3,3) | 3 (3,3) | 0.128 |
| BMI, median (IQR) | 23.9 (21.6, 26.0) | 22.68 (21.1, 25.0) | 0.067 |
| Nasal sIgA titers, GMT (95% CI) | 49.70 (43.68, 56.54) | 31.20 (15.38, 63.29) | 0.196 |
| Serum IgG (AU/mL), GMT (95% CI) | 159.59 (149.70, 170.14) | 145.32 (103.27, 204.50) | 0.586 |
| Memory B cells (%), median (IQR) | 1.11 (0.51, 1.89) | 1.13 (0.59, 1.57) | 0.981 |
| cTfh cells (%), median (IQR) | 0.64 (0.33, 1.11) | 0.61 (0.30, 1.12) | 0.847 |
| CD4^+^ T_CM_ cells (%), median (IQR) | 0.05 (0.01, 0.19) | 0.05 (0.03, 0.17) | 0.205 |
| CD4^+^ T_EM_ cells (%), median (IQR) | 0.82 (0.48, 1.24) | 0.65 (0.42, 1.33) | 0.684 |
| CD4^+^ T_EMRA_ cells (%), median (IQR) | 0.14 (0.07, 0.25) | 0.15 (0.08, 0.23) | 0.587 |
| CD8^+^ T_CM_ cells (%), median (IQR) | 0.03 (0.01, 0.06) | 0.04 (0.01, 0.05) | 0.599 |
| CD8^+^ T_EM_ cells (%), median (IQR) | 0.56 (0.28, 0.93) | 0.55 (0.39, 0.85) | 0.674 |
| CD8^+^ T_EMRA_ cells (%), median (IQR) | 0.44 (0.20, 0.74) | 0.45 (0.26, 0.83) | 0.221 |

Note: Data are presented as n (%) for categorical variables, median (IQR) for non-normally distributed continuous variables, and GMT (95% CI) for antibody titers. Abbreviations: IQR, interquartile range; CI, confidence interval; GMT, geometric mean titer; BMI, body mass index; sIgA, secretory immunoglobulin A; IgG, immunoglobulin G; cTfh, circulating follicular helper T cells; T_CM_, central memory T cells; T_EM_, effector memory T cells; T_EMRA_, terminally differentiated effector memory T cells.

**Table S3.** Baseline characteristics of the training, internal validation, and external validation cohorts.

| **Variables** | **Training cohort**  **(n = 400)** | **Internal validation cohort (n = 170)** | **External validation cohort**  **(n = 586)** | ***P* value** |
| --- | --- | --- | --- | --- |
| Reinfection, n (%) | 47 (11.8) | 19 (11.2) | 62 (10.6) | 0.847 |
| Age (years), median (IQR) | 60 (38, 71) | 53 (37, 68) | 65 (49, 73) | < 0.001 |
| Sex, n (%) |  |  |  | 0.090 |
| Male | 165 (41.2) | 80 (47.1) | 292 (49.8) |  |
| Female | 235 (58.8) | 90 (52.9) | 294 (50.2) |  |
| Smoking, n (%) |  |  |  | 0.461 |
| No | 322 (80.5) | 131 (77.1) | 457 (78.0) |  |
| Yes | 78 (19.5) | 39 (22.9) | 129 (22.0) |  |
| Drinking, n (%) |  |  |  | 0.001 |
| No | 314 (78.5) | 131 (77.1) | 406 (69.3) |  |
| Yes | 86 (21.5) | 39 (22.9) | 180 (30.7) |  |
| Diabetes, n (%) |  |  |  | 0.231 |
| No | 348 (87.0) | 156 (91.8) | 516 (88.1) |  |
| Yes | 52 (13.0) | 14 (8.2) | 70 (11.9) |  |
| BMI, median (IQR) | 23.5 (21.3, 25.6) | 23.9 (21.6, 26.0) | 24.2 (22.0, 26.3) | 0.002 |
| Number of COVID-19 infections, median (IQR) | 1 (1, 2) | 1 (1, 2) | 1 (1, 1) | < 0.001 |
| Number of COVID-19 vaccinations, median (IQR) | 3 (3, 4) | 3 (3, 4) | 3 (3, 3) | < 0.001 |
| Nasal sIgA titers, GMT (95% CI) | 37.47 (29.99, 46.81) | 34.42 (24.90, 47.58) | 67.05 (56.05, 80.19) | < 0.001 |
| Serum IgG (AU/mL), GMT (95% CI) | 189.19 (170.04, 210.49) | 209.98 (178.57, 246.92) | 131.22 (120.00, 143.50) | < 0.001 |
| Memory B cells (%), median (IQR) | 1.03 (0.46, 1.71) | 1.07 (0.61, 1.79) | 1.21 (0.53, 1.99) | 0.017 |
| cTfh cells (%), median (IQR) | 0.83 (0.44, 1.42) | 0.79 (0.38, 1.30) | 0.50 (0.26, 0.84) | < 0.001 |
| CD4^+^ T_CM_ cells (%), median (IQR) | 0.11 (0.02, 0.34) | 0.11 (0.02, 0.42) | 0.03 (0.01, 0.07) | < 0.001 |
| CD4^+^ T_EM_ cells (%), median (IQR) | 0.80 (0.47, 1.24) | 0.86 (0.51, 1.23) | 0.82 (0.49, 1.27) | 0.702 |
| CD4^+^ T_EMRA_ cells (%)，median (IQR) | 0.11 (0.05, 0.19) | 0.11 (0.05, 0.20) | 0.18 (0.08, 0.31) | < 0.001 |
| CD8^+^ T_CM_ cells (%)，median (IQR) | 0.03 (0.01, 0.06) | 0.03 (0.01, 0.08) | 0.03 (0.01, 0.06) | 0.058 |
| CD8^+^ T_EM_ cells (%)，median (IQR) | 0.53 (0.27, 0.88) | 0.55 (0.27, 0.88) | 0.57 (0.31, 0.95) | 0.345 |
| CD8^+^ T_EMRA_ cells (%)，median (IQR) | 0.50 (0.20, 0.79) | 0.37 (0.16, 0.70) | 0.43 (0.22, 0.71) | 0.053 |

Note: Data are presented as n (%) for categorical variables, median (IQR) for non-normally distributed continuous variables, and GMT (95% CI) for antibody titers. Abbreviations: IQR, interquartile range; CI, confidence interval; GMT, geometric mean titer; BMI, body mass index; sIgA, secretory immunoglobulin A; IgG, immunoglobulin G; cTfh, circulating follicular helper T cells; T_CM_, central memory T cells; T_EM_, effector memory T cells; T_EMRA_, terminally differentiated effector memory T cells.

**Table S4.** Feature-selection stability across 100 subsampling iterations (80% subsampling without replacement).

| Feature | Confirmed, n (%) | Tentative, n (%) | Rejected, n (%) |
| --- | --- | --- | --- |
| Nasal sIgA | 100 (100%) | 0 (0%) | 0 (0%) |
| CD4^+^ T_CM_ cells | 100 (100%) | 0 (0%) | 0 (0%) |
| BMI | 10 (10%) | 47 (47%) | 43 (43%) |
| CD8^+^ T_EMRA_ cells | 4 (4%) | 24 (24%) | 72 (72%) |
| Number of COVID-19 vaccinations | 2 (2%) | 11 (11%) | 87 (87%) |
| CD4^+^ T_EMRA_ cells | 2 (2%) | 8 (8%) | 90 (90%) |
| CD8^+^ T_CM_ cells | 0 (0%) | 8 (8%) | 92 (92%) |
| Serum IgG | 0 (0%) | 7 (7%) | 93 (93%) |
| Sex | 0 (0%) | 3 (3%) | 97 (97%) |
| Age | 0 (0%) | 3 (3%) | 97 (97%) |
| cTfh cells | 0 (0%) | 3 (3%) | 97 (97%) |
| Memory B cells | 0 (0%) | 2 (2%) | 98 (98%) |
| CD4^+^ T_EM_ cells | 0 (0%) | 2 (2%) | 98 (98%) |
| Drinking status | 0 (0%) | 1 (1%) | 99 (99%) |
| Smoking status | 0 (0%) | 0 (0%) | 100 (100%) |
| Diabetes | 0 (0%) | 0 (0%) | 100 (100%) |
| Number of prior COVID-19 infections | 0 (0%) | 0 (0%) | 100 (100%) |
| CD8^+^ T_EM_ cells | 0 (0%) | 0 (0%) | 100 (100%) |

Abbreviations: Values indicate the number and percentage of subsampling iterations in which each feature was classified by the Boruta algorithm as Confirmed, Tentative, or Rejected. Abbreviations: BMI, body mass index; sIgA, secretory immunoglobulin A; IgG, immunoglobulin G; cTfh, circulating follicular helper T cells; T_CM_, central memory T cells; T_EM,_ effector memory T cells; T_EMRA_, terminally differentiated effector memory T cells.

**Table S5.** Multivariable logistic regression analysis of nasal sIgA titers and CD4^+^ T_CM_ cells in relation to SARS-CoV-2 reinfection risk

| Characteristics | Crude model | | Model 1 | | Model 2 | |
| --- | --- | --- | --- | --- | --- | --- |
|  | OR  （95% CI） | *P* value | OR  （95% CI） | *P* value | OR（95%CI） | *P* value |
| Nasal sIgA titers | 0.995 (0.993-0.997) | <0.001 | 0.995 (0.993 -0.997) | <0.001 | 0.995 (0.993- 0.997) | <0.001 |
| CD4^+^ T_CM_ cells (%) | 0.447 (0.179 - 1.115) | 0.084 | 0.411 (0.163- 1.033) | 0.059 | 0.427 (0.167- 1.093) | 0.076 |

Note: ORs are presented with 95% CIs.

Crude model: Unadjusted.

Model 1: Adjusted for age.

Model 2: Adjusted for age, sex, prior SARS-CoV-2 infection history, COVID-19 vaccination history, alcohol consumption, smoking status, body mass index (BMI) and diabetes.

**Table S6.** Performance of machine learning models for predicting SARS-CoV-2 reinfection.

|  | **AUC (95% CI)** | **Accuracy** | **Sensitivity** | **Specificity** | **PPV** | **NPV** | **F1 score** |
| --- | --- | --- | --- | --- | --- | --- | --- |
| **Training cohort** |  |  |  |  |  |  |  |
| XGB | 0.914 (0.868-0.961) | 0.895 | 0.830 | 0.904 | 0.534 | 0.976 | 0.650 |
| ADB | 0.892 (0.832-0.951) | 0.930 | 0.809 | 0.946 | 0.667 | 0.974 | 0.731 |
| RF | 0.945 (0.921-0.969) | 0.810 | 0.979 | 0.788 | 0.380 | 0.996 | 0.548 |
| LR | 0.801 (0.734-0.867) | 0.782 | 0.723 | 0.790 | 0.315 | 0.955 | 0.439 |
| KNN | 0.957 (0.937-0.976) | 0.882 | 1.000 | 0.867 | 0.500 | 1.000 | 0.667 |
| GNB | 0.783 (0.711-0.855) | 0.818 | 0.723 | 0.830 | 0.362 | 0.958 | 0.482 |
| **Internal validation cohort** | |  |  |  |  |  |  |
| XGB | 0.835 (0.724-0.946) | 0.824 | 0.737 | 0.834 | 0.359 | 0.962 | 0.483 |
| ADB | 0.736 (0.610-0.862) | 0.906 | 0.526 | 0.954 | 0.588 | 0.941 | 0.556 |
| RF | 0.786 (0.641-0.931) | 0.841 | 0.737 | 0.854 | 0.389 | 0.963 | 0.509 |
| LR | 0.801 (0.705-0.897) | 0.688 | 0.842 | 0.669 | 0.242 | 0.971 | 0.376 |
| KNN | 0.805 (0.657-0.953) | 0.900 | 0.789 | 0.914 | 0.536 | 0.972 | 0.638 |
| GNB | 0.756 (0.636-0.876) | 0.835 | 0.684 | 0.854 | 0.371 | 0.956 | 0.481 |
| **External validation cohort** | |  |  |  |  |  |  |
| XGB | 0.996 (0.993-0.999) | 0.974 | 1.000 | 0.971 | 0.805 | 1.000 | 0.892 |
| ADB | 0.995 (0.991-0.999) | 0.991 | 1.000 | 0.990 | 0.925 | 1.000 | 0.961 |
| RF | 0.968 (0.946-0.989) | 0.944 | 0.935 | 0.945 | 0.667 | 0.992 | 0.779 |
| LR | 0.802 (0.740-0.863) | 0.732 | 0.790 | 0.725 | 0.254 | 0.967 | 0.384 |
| KNN | 0.949 (0.916-0.982) | 0.961 | 0.839 | 0.975 | 0.800 | 0.981 | 0.819 |
| GNB | 0.787 (0.719-0.856) | 0.814 | 0.790 | 0.817 | 0.338 | 0.971 | 0.473 |

**Note:** AUCs are presented with 95% confidence intervals. Accuracy, sensitivity, specificity, PPV, NPV, and F1 scores were calculated using the prespecified classification threshold. **Abbreviations:** AUC, area under the receiver operating characteristic curve; PPV, positive predictive value; NPV, negative predictive value; XGB, extreme gradient boosting; ADB, adaptive boosting; RF, random forest; LR, logistic regression; KNN, K-nearest neighbors; GNB, Gaussian naive Bayes.

**Table S7.** Performance of the XGB model in nested 5-fold cross-validation

| Model | Fold 1 AUC | Fold 2 AUC | Fold 3 AUC | Fold 4 AUC | Fold 5 AUC | Mean AUC ± SD |
| --- | --- | --- | --- | --- | --- | --- |
| XGB | 0.721 | 0.846 | 0.780 | 0.924 | 0.899 | 0.834 ± 0.084 |

Note: AUC values represent performance in the held-out outer folds of the nested 5-fold cross-validation procedure. SD, standard deviation; AUC, area under the receiver operating characteristic curve; XGB, extreme gradient boosting.
